# The Impact of Data Augmentations on Deep Learning-Based Marine Object Classification in Benthic Image Transects

**DOI:** 10.3390/s22145383

**Published:** 2022-07-19

**Authors:** Mingkun Tan, Daniel Langenkämper, Tim W. Nattkemper

**Affiliations:** Biodata Mining Group, Bielefeld University, P.O. Box 100131, 33501 Bielefeld, Germany; mtan@cebitec.uni-bielefeld.de (M.T.); dlangenk@cebitec.uni-bielefeld.de (D.L.)

**Keywords:** marine objects classification, underwater computer vision, deep learning, data augmentation

## Abstract

Data augmentation is an established technique in computer vision to foster the generalization of training and to deal with low data volume. Most data augmentation and computer vision research are focused on everyday images such as traffic data. The application of computer vision techniques in domains like marine sciences has shown to be not that straightforward in the past due to special characteristics, such as very low data volume and class imbalance, because of costly manual annotation by human domain experts, and general low species abundances. However, the data volume acquired today with moving platforms to collect large image collections from remote marine habitats, like the deep benthos, for marine biodiversity assessment and monitoring makes the use of computer vision automatic detection and classification inevitable. In this work, we investigate the effect of data augmentation in the context of taxonomic classification in underwater, i.e., benthic images. First, we show that established data augmentation methods (i.e., geometric and photometric transformations) perform differently in marine image collections compared to established image collections like the Cityscapes dataset, showing everyday traffic images. Some of the methods even decrease the learning performance when applied to marine image collections. Second, we propose new data augmentation combination policies motivated by our observations and compare their effect to those proposed by the AutoAugment algorithm and can show that the proposed augmentation policy outperforms the AutoAugment results for marine image collections. We conclude that in the case of small marine image datasets, background knowledge, and heuristics should sometimes be applied to design an effective data augmentation method.

## 1. Introduction

Underwater imaging with autonomous or remotely operated vehicles such as AUV (autonomous underwater vehicles [1]) or ROV (remotely operated vehicles [2]) allows visual assessments of large remote marine habitats through large image collections with $10^{2}$–$10^{4}$ images collected in one dive. One of the main purposes of these image collections is to provide valuable information about the biodiversity in marine life. To detect and classify species or morphotypes in these images, machine learning, has been proposed with some early promising results in the last decade obtained with pre-deep learning methods such as support vector machines (SVM) [3,4,5] or, more recently, using convolutional neural networks (CNN) [6,7,8,9,10,11,12,13,14,15,16,17,18,19]. The application of CNN for taxonomic classification problems in this marine image domain features some characteristics that separate this field of research from the majority of CNN applications in the context of human civilization (like traffic image classification/identification, quality control, observation, manufacturing). First, the process of image collection is very expensive as it involves costs for ship cruises, ROV/AUV hardware, operator personnel, advanced planning, maneuvering, and special camera equipment. Second, the number of domain experts that are required for collecting taxonomic labels to build training and test data is limited. In addition, the task of taxonomic classification in the images is very difficult as the domain experts usually have only one single image of an organism, in contrast to the traditional approach of collecting samples from the seafloor and intense visual inspection of the specimen in the laboratory. Another problem is that due to the natural structure of food webs and communities, some species are rather rare by nature and appear in just a few images and some species can be observed in hundreds of images, which leads to a strong class imbalance. Similar problems can be observed in aerial images collected for environmental monitoring. As a consequence, the field of marine image classification may sometimes require individual approaches to pattern recognition problems shaped by the characteristics listed above.

Data augmentation (DA) is a standard approach to overcome training problems caused by limitations in training data and over-fitting. In the case of image classification, the image augmentation algorithms can be broadly classified as deep learning approaches, for example, adversarial training [20], neural style transfer [21], feature space augmentation [22,23], generative adversarial networks (GAN) [24,25], meta-learning (AutoAugment [26], Smart Augmentation [27]), and basic image manipulation augmentations, for instance, geometric transformations (horizontal flipping, vertical flipping, random rotation, shearing), color space transformations (contrast modulation, brightness adjustment, hue variety), random erasing, mixing images [28,29], and kernel filters [30]. Deep learning approaches and basic image manipulation augmentations do not form a mutually exclusive dichotomy. In this work, we are mainly curious about the effectiveness of the most broadly used and readily available basic image manipulation operations in marine images. However, there is little literature available on methodological approaches to (i) select one or more data augmentation method(s) for a given image domain, or (ii) employ (or improve) the effect of DA in the marine image domain in particular. While single individual successful applications of DA have been reported already [14,17,19,31] also some examples have been reported on unsuccessful DA applications leading to decreasing performance [31,32,33].

Recently, a small number of concepts have been proposed for combinations of DA methods. Shorten and Khoshgoftaar [33] describes that it is important to consider the ‘safety’ of augmentation, and this is somewhat domain-dependent, providing a challenge for developing generalizable augmentation policies. Cubuk et al. have met the challenge of developing generalizable augmentation policies in their work, proposing the AutoAugment algorithm [26] to search for augmentation policies from a dataset automatically. Based on this work, Fast AutoAugment [34] optimized the search strategy, which speeds up the search time. One study was conducted by Shijie et al. [35], which compared the performance evaluation of some DA methods and their combinations on the CIFAR-10 and ImageNet datasets. They found four individual methods generally perform better than others, and some appropriate combinations of methods are slightly more effective than the individuals. However, although all these approaches provide highly valuable new methods, a domain-specific investigation of DA effectiveness is missing, especially for special domains like medical imaging, aerial images, digital microscopy, or -like in this case- benthic marine images.

As already explained, the marine imaging domain challenges deep learning applications with a permanent lack of annotated data. On the other hand, marine benthic images often are recorded with a downward-looking camera (sometimes referred to as “ortho-images”) that feature a higher degree of regularity (for instance, regarding illumination or camera—object distance). This can also increase the potential of machine learning-based classifications in benthic images when compared to everyday benchmark image data in computer vision documenting, for instance, different aspects of human activities (e.g., traffic scenes, social activities). Such images somehow constitute the mainstream in CNN application domains and show lesser irregularities in this regard due to changing weather, light condition, camera distance to the object, etc. Thus, we hypothesize that marine images may show special characteristics regarding the effectiveness of particular DA methods. These characteristics need to be thoroughly investigated as DA can be considered one of the most promising tools to overcome problems in missing or unbalanced training data in marine imaging. To show the effect of different DA methods in the context of deep learning classification in marine images, we first report results from exhaustive comparative experiments using single DA methods. Based on our findings, we propose different combinations of augmentation methods, referred to as augmentation policies, and can show a significant improvement for our marine-domain datasets.

## 2. Materials and Methods

### 2.1. Data Sets

To show the effect of different DA approaches, we conduct experiments with two marine-domain datasets. The Porcupine Abyssal Plane (PAP) which is in the northwest Atlantic Ocean, to the southwest of the United Kingdom [36,37,38] and the Clarion Clipperton Zone (CCZ) which is located in the Pacific Ocean and is known for its rich deposits of manganese nodules [39]. They are collected with AUVs in several 1000 m depths and show deep-sea benthos with different species. In addition to these two marine-domain datasets, we conduct a series of data augmentation experiments on the Cityscapes dataset [40], referred to as CSD, collected from annotated traffic videos in urban street scenes.

#### 2.1.1. PAP

**Figure 1:**
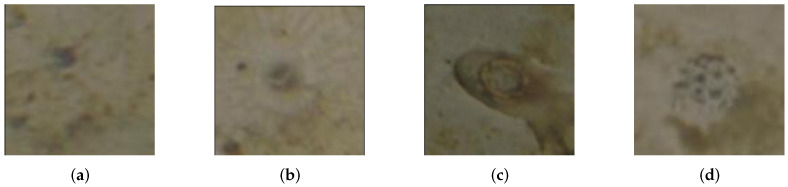
$\Gamma_{\mathrm{PAP}}$ dataset. (**a**) Ophiuroidea; (**b**) Cnidaria; (**c**) Amperima; (**d**) Foraminifera. Reproduced with permission from Henry Ruhl.

In this work, we choose the following four categories (see Figure 1) to form $\Gamma_{\mathrm{PAP}}$ for our experiments to ensure that a more trustworthy number of test samples are left.

**Figure 2:**
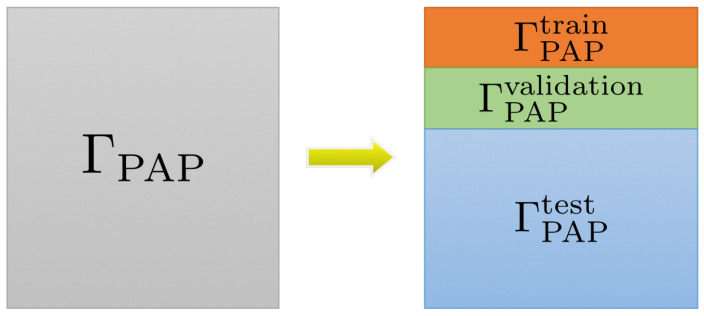
Dataset structure. The datasets were randomly subdivided into train, validation, and test set.

**Figure 3:**
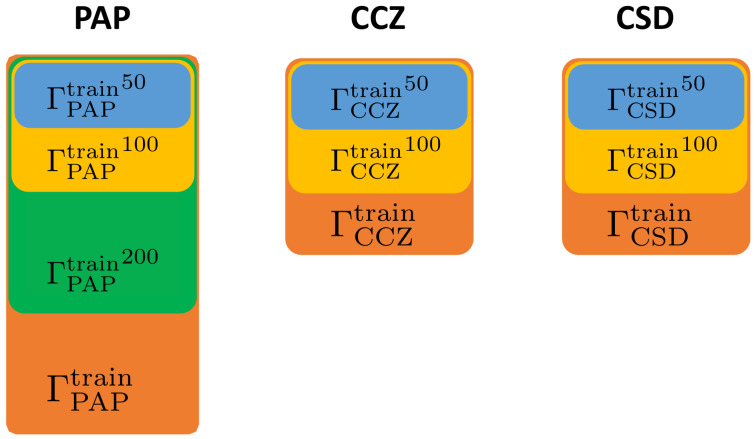
Construction of the training sets. Experiments were conducted using training sets of different sizes to investigate the influence of training set size on the impact of augmentation.

Figure 2 reveals the structure of the $\Gamma_{\mathrm{PAP}}$ dataset. For each class we randomly sample 300 images from $\Gamma_{\mathrm{PAP}}$ as $\Gamma_{\mathrm{PAP}}^{\mathrm{train}}$, 200 images as $\Gamma_{\mathrm{PAP}}^{\mathrm{validation}}$, and the rest as $\Gamma_{\mathrm{PAP}}^{\mathrm{test}}$. $\Gamma_{\mathrm{PAP}}$ = $\Gamma_{\mathrm{PAP}}^{\mathrm{train}}\cap \Gamma_{\mathrm{PAP}}^{\mathrm{validation}}\cap \Gamma_{\mathrm{PAP}}^{\mathrm{test}}$. Additionally, as shown in Figure 3 we sample 50, 100, and 200 images randomly from $\Gamma_{\mathrm{PAP}}^{\mathrm{train}}$ as $\Gamma_{\mathrm{PAP}}^{{\mathrm{train}}^{50}}$, $\Gamma_{\mathrm{PAP}}^{{\mathrm{train}}^{100}}$, $\Gamma_{\mathrm{PAP}}^{{\mathrm{train}}^{200}}$, respectively, to investigate the effect of train set size on DA performance. Sample sizes of each class are shown in Table 1.

#### 2.1.2. CCZ

**Figure 4:**
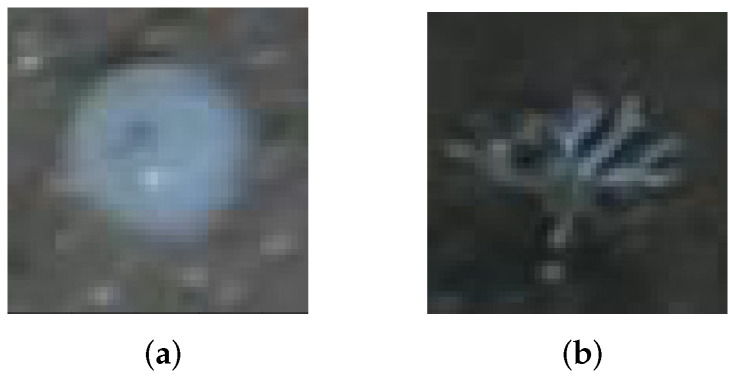
$\Gamma_{\mathrm{CCZ}}$ dataset. (**a**) Sponge; (**b**) Coral.

Similarly, we choose the two most abundant categories (shown in Figure 4) to form the $\Gamma_{\mathrm{CCZ}}$ dataset for the experiments in this work. Sample sizes of each class in the $\Gamma_{\mathrm{CCZ}}$ dataset are shown in Table 2.

#### 2.1.3. CSD

**Figure 5:**
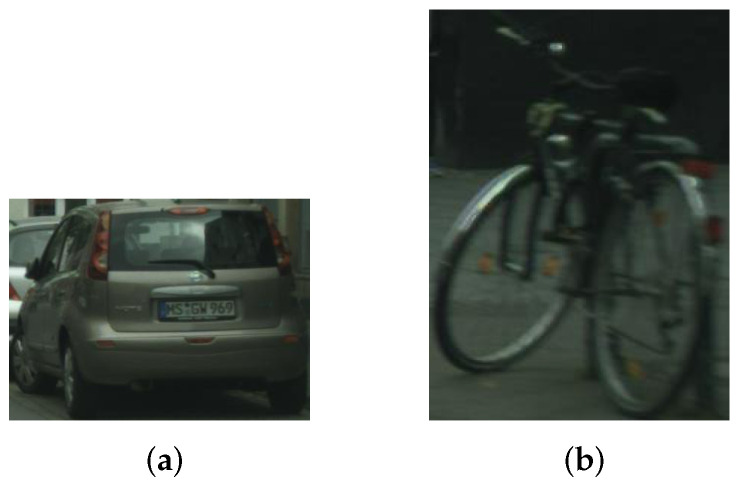
$\Gamma_{\mathrm{CSD}}$ dataset. (**a**) Car; (**b**) Bicycle.

We choose two classes (see Figure 5) from the vehicle group of the CSD to generate the dataset $\Gamma_{\mathrm{CSD}}$ (shown in Table 3) for our experiments in this work.

### 2.2. Model and Evaluation Criteria

**Figure 6:**
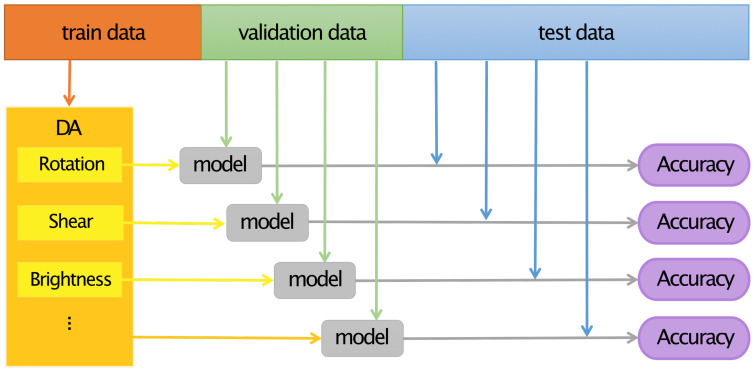
Flowchart of the work. Each DA method was applied to each train data and an individual model was trained and optimized using the validation data. The accuracy of the individually trained model was evaluated using the test data.

In this work, we use a MobileNet-v2 which is pre-trained on the ImageNet [41] to investigate several augmentation policies. Image data are resized to 224 px × 224 px and normalized based on the ImageNet dataset. We use Adam as the optimizer in our experiments and set a learning rate of 1 × 10${}^{-4}$ accompanied by a step decay with a step size of 1 and a gamma of 0.1. The loss function used in this work is cross-entropy loss. For each epoch, we perform a train and a validation phase, and compute the prediction accuracy $Acc_{e,j}=\frac{n_{e,j}}{N_{e,j}}$, where $n_{e,j}$ and $N_{e,j}$ stand for the number of correct predictions and the total number of samples in phase j∈{train,validation} of epoch *e*, respectively. In the test phase, since the sample sizes of each class in the test set are not consistent, we compute the prediction accuracy $Acc_{k}=\frac{n_{k}}{N_{k}}$ for each class, where $n_{k}$ and $N_{k}$ stand for the number of correct predictions for class *k* and the sample sizes of class *k* in the test set, respectively. In each experiment we record two trained-models corresponding to the highest $Acc_{e,\mathrm{train}}$ and the highest $Acc_{e,\mathrm{validation}}$ in all epochs and apply them to the test set separately. The two inference results are averaged to obtain the average accuracy $AA_{k}=\frac{1}{2}\left(Acc_{k}^{Acc_{e,\mathrm{train}}^{\mathrm{highest}}}+Acc_{k}^{Acc_{e,\mathrm{validation}}^{\mathrm{highest}}}\right)$ for each class. As the last step, we compute the mean average accuracy $mAA=\frac{1}{K}\sum_{k}AA_{k}$ with *K* the number of classes as the prediction accuracy in the test phase. The flowchart of our work is shown in Figure 6.

### 2.3. Methods

In this work, we investigate the data augmentations implemented in torchvision of PyTorch during the training progress. We describe the data augmentation in the way that a training image $X_{i}$ is given defined by $X_{i}=f\left(x_{i}\right)$ with $x_{i}$ as the original image with index *i* and f() as the transformation function executing the augmentation method. In this work we apply RandomRotation $f_{\mathrm{RR}}\left(x_{i},d\right)$ to rotate the image randomly within the angle range represented by *d*, RandomVerticalFlip $f_{\mathrm{RVF}}\left(x_{i},p\right)$ to vertically flip the given image randomly with a given probability *p*, RandomHorizontalFlip $f_{\mathrm{RHF}}\left(x_{i},p\right)$ to horizontally flip the given image randomly with a given probability *p*, RandomAffine $f_{\mathrm{RA}}\left(x_{i},t,s\right)$ to randomly affine transformation translate and shear of the image keeping center invariant according to the parameters *t* and *s*. Color transformations $f_{\mathrm{CT}}\left(x_{i},b,c,s,h\right)$ is used to randomly change the brightness, contrast, saturation, and hue of an image according to the values of parameters *b*, *c*, *s*, *h*, respectively.

We investigate the performance of DA methods on $\Gamma_{\mathrm{PAP}}$ and $\Gamma_{\mathrm{CCZ}}$, determining the four best-performing ones. We propose six DA combination policies and apply them to $\Gamma_{\mathrm{PAP}}$, $\Gamma_{\mathrm{CCZ}}$ and $\Gamma_{\mathrm{CSD}}$. To avoid randomness affecting the results during the experiments, we set a seed for fixing the following random number generators: CUDA, NumPy, Python, PyTorch, and cudnn.

## 3. Results

The experimental results are represented by the change of mAA of applying DA and without applying DA. We generate a heatmap for each experiment based on the change of mAA, with blue indicating positive increments and orange indicating decrease. The darker the color, the greater the change.

### 3.1. Experiment A: Performance of Data Augmentations on $\Gamma_{\mathrm{PAP}}$

We apply a series of DA methods and parameters to $\Gamma_{\mathrm{PAP}}^{{\mathrm{train}}^{50}}$, $\Gamma_{\mathrm{PAP}}^{{\mathrm{train}}^{100}}$, and $\Gamma_{\mathrm{PAP}}^{{\mathrm{train}}^{200}}$ to observe the performance of different DA and different parameters. The seed is fixed to 350 in all experiments to exclude the interference of the seed with the experiment. The results of $\Gamma_{\mathrm{PAP}}^{{\mathrm{train}}^{50}}$ and $\Gamma_{\mathrm{PAP}}^{{\mathrm{train}}^{200}}$ are shown in Table 4 and Table 5, and the results of $\Gamma_{\mathrm{PAP}}^{{\mathrm{train}}^{100}}$ are shown in Appendix A.

Table 4 reveals the performance of different DA methods and parameters when applied on $\Gamma_{\mathrm{PAP}}^{{\mathrm{train}}^{50}}$ with setting seed to 350. From this heatmap, we can see that almost all of the DA methods and parameters used in our experiments perform well. The best-performing DA methods and parameters on $\Gamma_{\mathrm{PAP}}^{{\mathrm{train}}^{50}}$ are RandomRotation with a parameter of 100°, Contrast with a parameter of 5, Brightness with a parameter of 2, and Shear with a parameter of (−30°, 30°, −30°, 30°). By applying these four, a significant improvement can be achieved on $\Gamma_{\mathrm{PAP}}^{{\mathrm{train}}^{50}}$.

When increasing the number of training images to 200 per class, we can see from Table 5 that the effect of DA on the improvement of average accuracy further diminishes. The best-performing four DA methods are still the same and the best-performing parameters of RandomRotation, Brightness, Contrast, and Shear are 170°, 1.9, 4.5, (−40°, 40°, −40°, 40°), respectively.

In Experiment A, we use $\Gamma_{\mathrm{PAP}}^{\mathrm{validation}}$ and $\Gamma_{\mathrm{PAP}}^{\mathrm{test}}$ as validation set and test set. The four best-performing DA methods are RandomRotation, Brightness, Contrast, and Shear, regardless of whether the training set is $\Gamma_{\mathrm{PAP}}^{{\mathrm{train}}^{50}}$, $\Gamma_{\mathrm{PAP}}^{{\mathrm{train}}^{100}}$, or $\Gamma_{\mathrm{PAP}}^{{\mathrm{train}}^{200}}$. As the size of training samples increases, the best-performing parameters of the DA vary. The increment of mAA shows an almost proportional trend to the magnitude of the parameters of RandomRotation, Brightness, Contrast, and Saturation. The performance of Shear reveals that the parameters that introduce more deformation can yield a better augmentation effect. However, it can be shearing in one direction at a bigger angle, or shearing in two directions at one angle.

We also conduct experiments on $\Gamma_{\mathrm{PAP}}^{{\mathrm{train}}^{50}}$ when setting the seed to 3500, finding that the heatmap shows a similar trend to that with the seed set to 350. The results are shown in Appendix B.

### 3.2. Experiment B: Performance of Data Augmentations on $\Gamma_{\mathrm{CCZ}}$

In Experiment B, we apply the same DA methods and parameters to $\Gamma_{\mathrm{CCZ}}$ to verify whether the observations we obtained in Experiment A can be seen on another marine-domain dataset as well. The experimental results with setting seed to 350 are shown in Table 6 and Table 7, and the results of $\Gamma_{\mathrm{CCZ}}^{{\mathrm{train}}^{50}}$ with setting seed to 3500 are shown in Appendix B.

The heatmap shown in Table 6 presents the performance of different DA methods and parameters when applied on $\Gamma_{\mathrm{CCZ}}^{{\mathrm{train}}^{50}}$ with setting seed to 350. We can find that the best-performing DA methods on $\Gamma_{\mathrm{CCZ}}^{{\mathrm{train}}^{50}}$ are the same as the observations from Experiment A, which are RandomRotation, Brightness, Contrast, and Shear. On $\Gamma_{\mathrm{CCZ}}^{{\mathrm{train}}^{50}}$, RandomRotation shows the best results with parameter 150°. Brightness, Contrast, and Shear work best with parameters 1.9, 4.5, and (−40°, 40°, −40°, 40°), respectively.

Table 7 shows the performance of different DA methods and parameters when applied on $\Gamma_{\mathrm{CCZ}}^{{\mathrm{train}}^{100}}$ with setting seed to 350. We increase the number of training data from 50 even to 100 per class. The effect of DA methods, except for Saturation, becomes weaker or has a negative effect as the number of training samples is increased, which is the same as shown in Table 5. The four most effective DA methods are still RandomRotation, Brightness, Contrast, and Shear, whose best-performing parameters are 170°, 2, 5, and 40°, respectively.

Experiment B investigates the effect of different DA methods and different parameters on $\Gamma_{\mathrm{CCZ}}$ with different training set sizes and shows similar results to the observations on $\Gamma_{\mathrm{PAP}}$. We find that RandomRotation, Contrast, Brightness, and Shear are always the four best-performing DA methods on both $\Gamma_{\mathrm{PAP}}$ and $\Gamma_{\mathrm{CCZ}}$ marine-domain dataset. The effect of RandomRotation, Brightness, and Contrast becomes more significant as the parameters increase. Similarly, as the amount of training data is increased, almost all DA methods’ effects are diminished.

### 3.3. Experiment C: Performance of Data Augmentations on $\Gamma_{\mathrm{CSD}}$

We conduct research on $\Gamma_{\mathrm{CSD}}$ that is different from the marine domain to demonstrate that the effect of DA is domain-dependent. The seed is set to 350 in Experiment C, and the experimental results of $\Gamma_{\mathrm{CSD}}^{{\mathrm{train}}^{50}}$ and $\Gamma_{\mathrm{CSD}}^{{\mathrm{train}}^{150}}$ are shown in Table 8 and Table 9. The results of $\Gamma_{\mathrm{CSD}}^{{\mathrm{train}}^{100}}$ are shown in Appendix A.

Table 8 illustrates the effect of different DA methods and different parameters on the average accuracy improvement of $\Gamma_{\mathrm{CSD}}^{{\mathrm{train}}^{50}}$. From this heatmap, we can see that RandomRotation no longer works as well as it did on the marine-domain datasets, and only when the parameters are small does it improve the average accuracy a little. Similarly, Shear with parameters of big degrees decreases the mAA. In addition, RandomVerticalFlip is also no longer suitable for this dataset.

When training samples are supplemented to 150 per class, we can see from Table 9 that RandomRotation with a parameter of big degrees and RandomVerticalFlip still have a negative impact. The effect of Brightness, Contrast, and Saturation on $\Gamma_{\mathrm{CSD}}^{{\mathrm{train}}^{150}}$ performs well with the value of parameters increasing, which is similar to that on $\Gamma_{\mathrm{PAP}}^{{\mathrm{train}}^{50}}$ and $\Gamma_{\mathrm{CCZ}}^{{\mathrm{train}}^{50}}$.

Experiment C investigated the performance of different DA methods and parameters on $\Gamma_{\mathrm{CSD}}$ with different training set sizes. Overall, Saturation shows an effect almost proportional to the value of parameters on all three datasets. The performance of Contrast and Brightness improve with increasing training data size. RandomRotation can slightly increase mAA with parameters smaller than 10°, but have increasingly negative effects on mAA as the parameters become larger. RandomHorizontalFlip and Hue can slightly improve mAA, while RandomVerticalFlip and Translate almost always reduce mAA. The effect of Shear is no longer as shown in Experiment A and Experiment B, showing a negative effect at large deformation angles.

**Figure 7:**
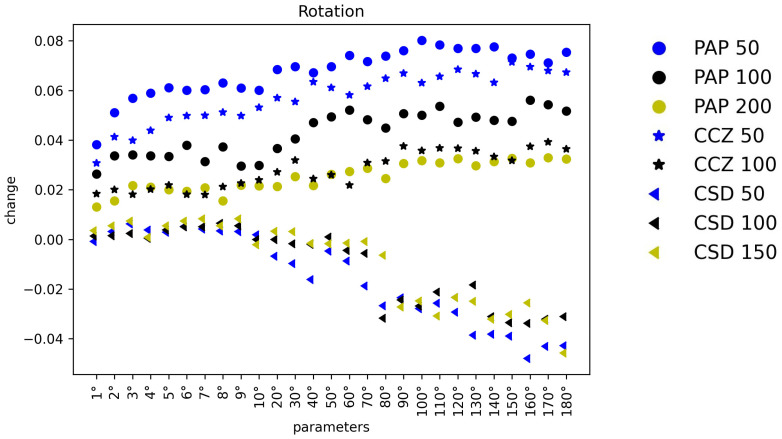

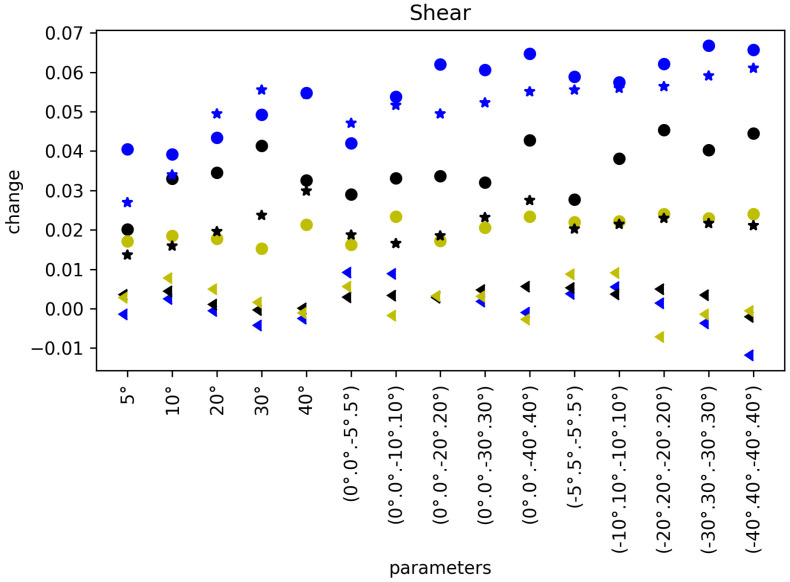
Impacts comparison of Rotation and Shear. The figure reveals the impacts of the different parameters of Rotation and Shear on $\Gamma_{\mathrm{PAP}}$, $\Gamma_{\mathrm{CCZ}}$, and $\Gamma_{\mathrm{CSD}}$ classification performance improvement.

**Figure 8:**
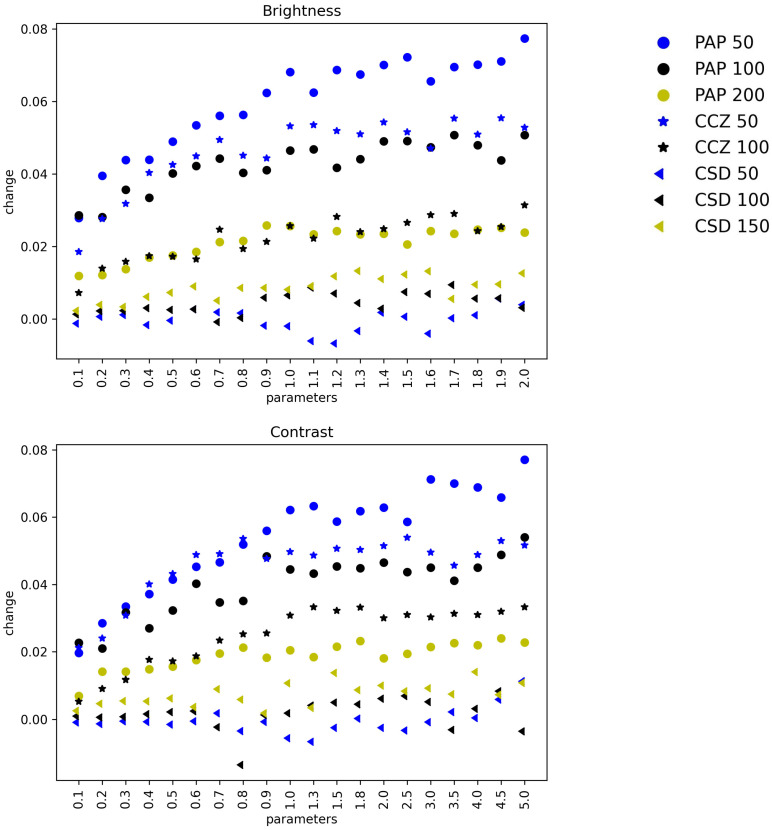

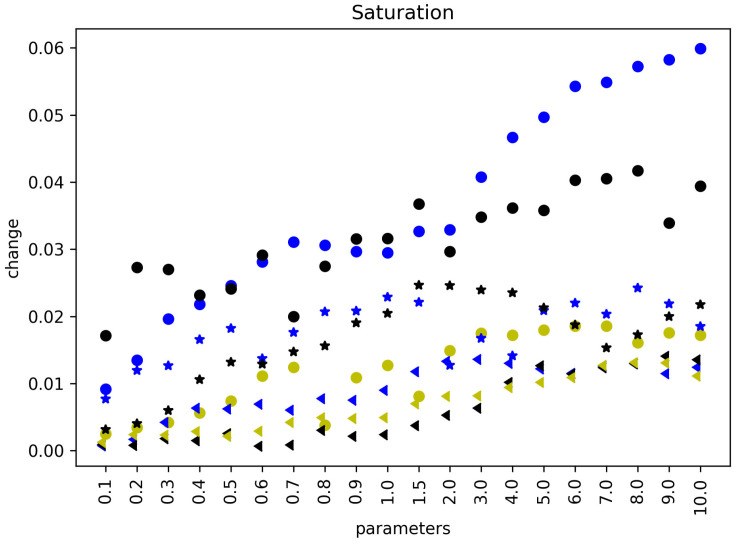
Impacts comparison of Brightness, Contrast and Saturation. The impacts of Brightness, Contrast and Saturation on improving the classification performance on $\Gamma_{\mathrm{PAP}}$, $\Gamma_{\mathrm{CCZ}}$, and $\Gamma_{\mathrm{CSD}}$ are plotted in the figure.

To compare the different DA methods’ impact on the three different datasets more intuitively, the percentage change of classification mAA is plotted for different parameters, which are shown in Figure 7 and Figure 8 and Appendix C. In each plot, the *x*-axis represents parameters, the *y*-axis represents the change of mAA, and circles, stars, triangle stand for $\Gamma_{\mathrm{PAP}}$, $\Gamma_{\mathrm{CCZ}}$, and $\Gamma_{\mathrm{CSD}}$, respectively.

### 3.4. Experiment D: Data Augmentation Policies

According to the above experimental results, the four best-performing DA methods on both $\Gamma_{\mathrm{PAP}}$ and $\Gamma_{\mathrm{CCZ}}$ datasets are RandomRotation, Contrast, Brightness, and Shear. In most cases, the best performances are shown when the parameter of RandomRotation is around 150°, the parameters of Contrast and Brightness are around 2 and 5, respectively, and the parameters of Shear are those that produce larger deformations (we experiment with the parameters 40). To verify the effect of the combination of these DA methods, we proposed DA combination policies in Table 10, where the function of RBC_1 indicates that RandomRotation 150°, Brightness 1.9, and Contrast 5 are applied to the training images successively.

The performance of our policies on $\Gamma_{\mathrm{PAP}}$, $\Gamma_{\mathrm{CCZ}}$, and $\Gamma_{\mathrm{CSD}}$ are shown in Table 11, where AA_IP and AA_CP represent ImageNet policy and CIFAR-10 policy proposed by AutoAugment [26], respectively. It shows that all our policies trained on $\Gamma_{\mathrm{PAP}}^{{\mathrm{train}}^{50}}$, $\Gamma_{\mathrm{PAP}}^{{\mathrm{train}}^{100}}$, $\Gamma_{\mathrm{PAP}}^{{\mathrm{train}}^{200}}$, $\Gamma_{\mathrm{PAP}}^{\mathrm{train}}$, and $\Gamma_{\mathrm{CCZ}}^{{\mathrm{train}}^{50}}$ can outperform AA_IP and AA_CP policies which are proposed by AutoAugment [26]. RBC_3, RBC_5, and RBCS trained on $\Gamma_{\mathrm{CCZ}}^{{\mathrm{train}}^{100}}$ and $\Gamma_{\mathrm{CCZ}}^{\mathrm{train}}$ can also outperform AA_IP and AA_CP. In contrast to the effect of our policies on $\Gamma_{\mathrm{PAP}}$ and $\Gamma_{\mathrm{CCZ}}$, these policies have a negative effect on the $\Gamma_{\mathrm{CSD}}$ dataset. Here the AutoAugment policies outperform our policies.

## 4. Discussion

In this paper, we could show a clear domain dependence for the application of augmentation. While experiments A and B applying augmentation to marine data show similar results, experiment C applied to the more established everyday traffic data shows different trends. The same observation applies to experiment D when comparing auto augmentation policies fit on everyday data (ImageNet and CIFAR-10) on the one hand with our manual combination policies on the other hand. Here the AutoAugment policies work best on the traffic data while leading to results inferior to the policies proposed by our experiments (RBC${\_}_{*}$). From Experiment A (marine), we can find that the effect of DA diminishes as the number of samples in the training set increases. This is because the additional training images allow the model to learn more features, which also indicates that DA is an effective way to address the lack of training data. The results of Experiment A and Experiment B (marine) indicate that for the marine domain, with increasing training sample size and different parameter choices, some DA methods show the possibility of decreasing mAA (e.g., flip, translate), but RandomRotation, Brightness, Contrast, and Shear always show good results. This may be due to the natural variation regarding the orientation and position of the underwater target objects relative to the camera. Besides, the light during underwater data acquisition has a significant effect on the image data. Overall, experiments A and B show similar trends. However, due to the insufficient sample size of $\Gamma_{\mathrm{CCZ}}^{\mathrm{test}}$, the results of Experiment B are not as reliable as those of Experiment A.

In Experiment C (traffic), the effect of RandomRotation, RandomVerticalFlip, and Shear have a significantly different effect than in Experiment A and Experiment B, by often even decreasing the performance. This is likely caused by the fact that a VerticalFlip or a Rotation at a large angle is unrealistic under the urban traffic domain. We can find that on the $\Gamma_{\mathrm{CSD}}$ dataset, the color transformations perform better than the geometric transformations.

From Table 11, we can see that the performance of our policies can reach or outperform that of transferring the policies proposed by AutoAugment [26] to marine data. However, our policies have a negative effect on the traffic dataset. As can be seen in Table 4 and Table 5, and the experimental result of $\Gamma_{\mathrm{PAP}}^{{\mathrm{train}}^{100}}$ in Appendix A, the maximum increments of mAA are 8.02%, 5.61%, and 3.29% on $\Gamma_{\mathrm{PAP}}^{{\mathrm{train}}^{50}}$, $\Gamma_{\mathrm{PAP}}^{{\mathrm{train}}^{100}}$, and $\Gamma_{\mathrm{PAP}}^{{\mathrm{train}}^{200}}$ datasets, respectively. After applying our policies, the increments of mAA in the $\Gamma_{\mathrm{PAP}}^{\mathrm{train}}$ dataset all exceed the maximum increments of applying a single DA method, while AA-IP and AA-CP are not as effective as the best single DA method on $\Gamma_{\mathrm{PAP}}^{{\mathrm{train}}^{100}}$ and $\Gamma_{\mathrm{PAP}}^{{\mathrm{train}}^{200}}$. A similar performance can be observed on $\Gamma_{\mathrm{CCZ}}$. It indicates that for our marine-domain data, RandomRotation works very well. However, they have the opposite effect on traffic-domain dataset $\Gamma_{\mathrm{CSD}}$, revealing that DA methods have a strong domain dependency.

## 5. Conclusions

We have shown that in our work, we could observe a clear difference in the effects of DA applied to our domain-specific marine dataset or the more established everyday urban traffic dataset. Therefore we propose to use DA with lower expectations, especially when applied to image domains that differ from the mainstream image domains CNNs are applied to. As we can show good results with a customized DA policy, we conclude that DA can definitely be the tool of choice, especially for small training sets, but increased efforts are required to make data augmentation more adaptive and domain aware.

## Figures and Tables

**Table 1:** Sample sizes of each category per subset in the $\Gamma_{\mathrm{PAP}}$ dataset.

$\Gamma_{\mathrm{PAP}}$	Ophiuroidea	Cnidaria	Amperima	Foraminifera
$\Gamma_{\mathrm{PAP}}^{{\mathrm{train}}^{50}}$	50	50	50	50
$\Gamma_{\mathrm{PAP}}^{{\mathrm{train}}^{100}}$	100	100	100	100
$\Gamma_{\mathrm{PAP}}^{{\mathrm{train}}^{200}}$	200	200	200	200
$\Gamma_{\mathrm{PAP}}^{\mathrm{train}}$	300	300	300	300
$\Gamma_{\mathrm{PAP}}^{\mathrm{validation}}$	200	200	200	200
$\Gamma_{\mathrm{PAP}}^{\mathrm{test}}$	8883	8861	5202	2132

**Table 2:** Sample sizes of category per subset in the $\Gamma_{\mathrm{CCZ}}$ dataset.

$\Gamma_{\mathrm{CCZ}}$	Sponge	Coral
$\Gamma_{\mathrm{CCZ}}^{{\mathrm{train}}^{50}}$	50	50
$\Gamma_{\mathrm{CCZ}}^{{\mathrm{train}}^{100}}$	100	100
$\Gamma_{\mathrm{CCZ}}^{\mathrm{train}}$	150	150
$\Gamma_{\mathrm{CCZ}}^{\mathrm{validation}}$	150	150
$\Gamma_{\mathrm{CCZ}}^{\mathrm{test}}$	1236	700

**Table 3:** Sample sizes of each category per subset in the $\Gamma_{\mathrm{CSD}}$ dataset.

$\Gamma_{\mathrm{CSD}}$	Car	Bicycle
$\Gamma_{\mathrm{CSD}}^{{\mathrm{train}}^{50}}$	50	50
$\Gamma_{\mathrm{CSD}}^{{\mathrm{train}}^{100}}$	100	100
$\Gamma_{\mathrm{CSD}}^{\mathrm{train}}$	150	150
$\Gamma_{\mathrm{CSD}}^{\mathrm{validation}}$	200	200
$\Gamma_{\mathrm{CSD}}^{\mathrm{test}}$	24,371	3208

**Table 4:** Performance of DA methods on $\Gamma_{\mathrm{PAP}}^{{\mathrm{train}}^{50}}$. The impact of DA methods and parameters on classification performance for $\Gamma_{\mathrm{PAP}}^{{\mathrm{train}}^{50}}$ is revealed. The displayed percentage values describe the increase/decrease of mAA in percent. The value is color-coded from blue (increase in accuracy) over white (no effect) to orange (decrease in accuracy).

**Random Rotation**		**Brightness**		**Contrast**		**Saturation**		**Random** **VerticalFlip**		**Random** **HorizontalFlip**	
1°	3.82%	0.1	2.79%	0.1	1.97%	0.1	0.92%	0.1	2.68%	0.1	1.65%
2°	5.11%	0.2	3.95%	0.2	2.85%	0.2	1.35%	0.2	3.15%	0.2	1.93%
3°	5.69%	0.3	4.38%	0.3	3.34%	0.3	1.96%	0.3	3.24%	0.3	1.95%
4°	5.89%	0.4	4.40%	0.4	3.71%	0.4	2.18%	0.4	3.51%	0.4	1.99%
5°	6.11%	0.5	4.90%	0.5	4.15%	0.5	2.46%	0.5	3.57%	0.5	2.11%
6°	6.01%	0.6	5.35%	0.6	4.53%	0.6	2.81%	0.6	3.66%	0.6	2.14%
7°	6.03%	0.7	5.61%	0.7	4.66%	0.7	3.11%	0.7	3.57%	0.7	2.11%
8°	6.30%	0.8	5.64%	0.8	5.19%	0.8	3.06%	0.8	3.31%	0.8	1.80%
9°	6.10%	0.9	6.24%	0.9	5.59%	0.9	2.97%	0.9	3.12%	0.9	1.56%
10°	6.01%	1	6.82%	1	6.22%	1	2.95%	1	0.34%	1	–1.16%
20°	6.85%	1.1	6.25%	1.3	6.33%	1.5	3.27%	Shear			
30°	6.97%	1.2	6.87%	1.5	5.87%	2	3.29%				
40°	6.71%	1.3	6.75%	1.8	6.18%	3	4.08%	5°			4.05%
50°	6.96%	1.4	7.01%	2	6.29%	4	4.67%	10°			3.92%
60°	7.41%	1.5	7.22%	2.5	5.86%	5	4.97%	20°			4.34%
70°	7.17%	1.6	6.56%	3	7.13%	6	5.43%	30°			4.93%
80°	7.39%	1.7	6.95%	3.5	7.00%	7	5.49%	40°			5.48%
90°	7.61%	1.8	7.01%	4	6.88%	8	5.72%	(0°, 0°, −5°, 5°)			4.20%
100°	8.02%	1.9	7.11%	4.5	6.59%	9	5.83%	(0°, 0°, −10°, 10°)			5.38%
110°	7.84%	2	7.74%	5	7.70%	10	5.99%	(0°, 0°, −20°, 20°)			6.20%
120°	7.70%	Hue		Translate				(0°,0°, −30°, 30°)			6.06%
130°	7.69%							(0°, 0°, −40°, 40°)			6.47%
140°	7.76%	0.1	4.17%	(0.1, 0.1)			5.59%	(−5°, 5°, −5°, 5°)			5.89%
150°	7.31%	0.2	5.13%	(0.2, 0.2)			4.72%	(−10°, 10°, −10°, 10°)			5.75%
160°	7.47%	0.3	4.86%	(0.3, 0.3)			4.21%	(−20°, 20°, −20°, 20°)			6.22%
170°	7.11%	0.4	4.52%	(0.4, 0.4)			2.53%	(−30°, 30°, −30°, 30°)			6.68%
180°	7.55%	0.5	4.70%	(0.5, 0.5)			1.40%	(−40°, 40°, −40°, 40°)			6.58%

**Table 5:** Performance of DA methods on $\Gamma_{\mathrm{PAP}}^{{\mathrm{train}}^{200}}$. The DA methods and parameters impact on $\Gamma_{\mathrm{PAP}}^{{\mathrm{train}}^{200}}$ classification performance is shown. The displayed percentage values describe the increase/decrease of mAA in percent. The value is color-coded from blue (increase in accuracy) over white (no effect) to orange (decrease in accuracy).

**Random Rotation**		**Brightness**		**Contrast**		**Saturation**		**Random** **VerticalFlip**		**Random** **HorizontalFlip**	
1°	1.30%	0.1	1.19%	0.1	0.69%	0.1	0.25%	0.1	0.94%	0.1	0.77%
2°	1.55%	0.2	1.22%	0.2	1.41%	0.2	0.34%	0.2	0.81%	0.2	0.92%
3°	2.18%	0.3	1.38%	0.3	1.41%	0.3	0.42%	0.3	1.01%	0.3	0.80%
4°	2.11%	0.4	1.70%	0.4	1.48%	0.4	0.57%	0.4	0.86%	0.4	0.88%
5°	2.01%	0.5	1.75%	0.5	1.56%	0.5	0.74%	0.5	1.31%	0.5	0.79%
6°	1.94%	0.6	1.85%	0.6	1.76%	0.6	1.11%	0.6	0.89%	0.6	0.82%
7°	2.08%	0.7	2.13%	0.7	1.95%	0.7	1.24%	0.7	0.77%	0.7	0.74%
8°	1.55%	0.8	2.15%	0.8	2.12%	0.8	0.38%	0.8	0.56%	0.8	0.70%
9°	2.18%	0.9	2.59%	0.9	1.82%	0.9	1.09%	0.9	0.78%	0.9	0.62%
10°	2.15%	1	2.57%	1	2.05%	1	1.27%	1	−0.43%	1	−0.43%
20°	2.14%	1.1	2.34%	1.3	1.84%	1.5	0.81%	Shear			
30°	2.53%	1.2	2.43%	1.5	2.15%	2	1.49%				
40°	2.17%	1.3	2.34%	1.8	2.32%	3	1.75%	5°			1.70%
50°	2.62%	1.4	2.35%	2	1.81%	4	1.72%	10°			1.85%
60°	2.74%	1.5	2.06%	2.5	1.94%	5	1.80%	20°			1.78%
70°	2.86%	1.6	2.43%	3	2.15%	6	1.85%	30°			1.53%
80°	2.45%	1.7	2.35%	3.5	2.26%	7	1.86%	40°			2.13%
90°	3.06%	1.8	2.47%	4	2.19%	8	1.61%	(0°, 0°, −5°, 5°)			1.63%
100°	3.17%	1.9	2.52%	4.5	2.40%	9	1.76%	(0°, 0°, −10°, 10°)			2.34%
110°	3.09%	2	2.39%	5	2.28%	10	1.72%	(0°, 0°, −20°, 20°)			1.72%
120°	3.25%	Hue		Translate				(0°, 0°, −30°, 30°)			2.06%
130°	2.96%							(0°, 0°, −40°, 40°)			2.33%
140°	3.14%	0.1	1.45%	(0.1, 0.1)			2.00%	(−5°, 5°, −5°, 5°)			2.20%
150°	3.27%	0.2	1.61%	(0.2, 0.2)			1.67%	(−10°, 10°, −10°, 10°)			2.22%
160°	3.08%	0.3	1.94%	(0.3, 0.3)			1.57%	(−20°, 20°, −20°, 20°)			2.40%
170°	3.29%	0.4	1.73%	(0.4, 0.4)			0.69%	(−30°, 30°, −30°, 30°)			2.29%
180°	3.23%	0.5	1.72%	(0.5, 0.5)			0.21%	(−40°, 40°, −40°, 40°)			2.40%

**Table 6:** Performance of DA methods on $\Gamma_{\mathrm{CCZ}}^{{\mathrm{train}}^{50}}$. The impact of different DA methods and parameters on $\Gamma_{\mathrm{CCZ}}^{{\mathrm{train}}^{50}}$ classification performance is displayed. The displayed percentage values describe the increase/decrease of mAA in percent. The value is color-coded from blue (increase in accuracy) over white (no effect) to orange (decrease in accuracy).

**Random Rotation**		**Brightness**		**Contrast**		**Saturation**		**Random** **VerticalFlip**		**Random** **HorizontalFlip**	
1°	3.07%	0.1	1.86%	0.1	2.12%	0.1	0.77%	0.1	2.93%	0.1	2.27%
2°	4.13%	0.2	2.77%	0.2	2.40%	0.2	1.20%	0.2	3.05%	0.2	2.67%
3°	3.99%	0.3	3.18%	0.3	3.08%	0.3	1.26%	0.3	3.24%	0.3	2.60%
4°	4.39%	0.4	4.03%	0.4	4.01%	0.4	1.66%	0.4	3.01%	0.4	2.53%
5°	4.91%	0.5	4.26%	0.5	4.32%	0.5	1.82%	0.5	2.80%	0.5	2.60%
6°	4.98%	0.6	4.50%	0.6	4.88%	0.6	1.37%	0.6	3.04%	0.6	2.85%
7°	5.00%	0.7	4.94%	0.7	4.91%	0.7	1.76%	0.7	3.01%	0.7	2.78%
8°	5.12%	0.8	4.51%	0.8	5.36%	0.8	2.07%	0.8	3.15%	0.8	2.65%
9°	4.97%	0.9	4.44%	0.9	4.77%	0.9	2.08%	0.9	2.63%	0.9	2.31%
10°	5.31%	1	5.32%	1	4.97%	1	2.29%	1	−0.93%	1	−0.99%
20°	5.70%	1.1	5.35%	1.3	4.86%	1.5	2.21%	Shear			
30°	5.54%	1.2	5.19%	1.5	5.06%	2	1.27%				
40°	6.35%	1.3	5.10%	1.8	5.03%	3	1.67%	5°			2.70%
50°	6.11%	1.4	5.43%	2	5.15%	4	1.42%	10°			3.39%
60°	5.81%	1.5	5.16%	2.5	5.39%	5	2.09%	20°			4.95%
70°	6.17%	1.6	4.71%	3	4.95%	6	2.20%	30°			5.55%
80°	6.48%	1.7	5.53%	3.5	4.56%	7	2.03%	40°			5.44%
90°	6.69%	1.8	5.09%	4	4.88%	8	2.43%	(0°, 0°, −5°, 5°)			4.71%
100°	6.31%	1.9	5.54%	4.5	5.30%	9	2.19%	(0°, 0°, −10°, 10°)			5.16%
110°	6.57%	2	5.28%	5	5.16%	10	1.85%	(0°, 0°, −20°, 20°)			4.95%
120°	6.85%	Hue		Translate				(0°, 0°, −30°, 30°)			5.23%
130°	6.66%							(0°, 0°, −40°, 40°)			5.51%
140°	6.31%	0.1	3.18%	(0.1, 0.1)			4.98%	(−5°, 5°, −5°, 5°)			5.55%
150°	7.14%	0.2	3.96%	(0.2, 0.2)			4.19%	(−10°, 10°, −10°, 10°)			5.60%
160°	6.95%	0.3	3.98%	(0.3, 0.3)			3.76%	(−20°, 20°, −20°, 20°)			5.64%
170°	6.80%	0.4	3.48%	(0.4, 0.4)			3.09%	(−30°, 30°, −30°, 30°)			5.91%
180°	6.73%	0.5	2.95%	(0.5, 0.5)			−0.15%	(−40°, 40°, −40°, 40°)			6.11%

**Table 7:** Performance of DA methods on $\Gamma_{\mathrm{CCZ}}^{{\mathrm{train}}^{100}}$. The effect of different DA methods and different parameters on $\Gamma_{\mathrm{CCZ}}^{{\mathrm{train}}^{100}}$ classification mAA improvement is shown. The displayed percentage values describe the increase/decrease of mAA in percent. The value is color-coded from blue (increase in accuracy) over white (no effect) to orange (decrease in accuracy).

**Random Rotation**		**Brightness**		**Contrast**		**Saturation**		**Random** **VerticalFlip**		**Random** **HorizontalFlip**	
1°	1.84%	0.1	0.73%	0.1	0.53%	0.1	0.32%	0.1	1.50%	0.1	0.92%
2°	2.01%	0.2	1.40%	0.2	0.91%	0.2	0.40%	0.2	1.29%	0.2	0.92%
3°	1.81%	0.3	1.59%	0.3	1.18%	0.3	0.60%	0.3	1.26%	0.3	0.99%
4°	2.02%	0.4	1.74%	0.4	1.76%	0.4	1.06%	0.4	1.25%	0.4	1.26%
5°	2.18%	0.5	1.73%	0.5	1.72%	0.5	1.32%	0.5	1.10%	0.5	1.14%
6°	1.82%	0.6	1.65%	0.6	1.87%	0.6	1.29%	0.6	1.17%	0.6	1.24%
7°	1.80%	0.7	2.47%	0.7	2.34%	0.7	1.48%	0.7	0.95%	0.7	1.20%
8°	2.12%	0.8	1.93%	0.8	2.52%	0.8	1.56%	0.8	0.84%	0.8	0.90%
9°	2.26%	0.9	2.13%	0.9	2.55%	0.9	1.91%	0.9	0.73%	0.9	0.87%
10°	2.39%	1	2.57%	1	3.08%	1	2.05%	1	−1.07%	1	−0.19%
20°	2.71%	1.1	2.22%	1.3	3.33%	1.5	2.46%	Shear			
30°	3.19%	1.2	2.83%	1.5	3.22%	2	2.46%				
40°	2.45%	1.3	2.41%	1.8	3.32%	3	2.40%	5°			1.36%
50°	2.60%	1.4	2.49%	2	3.00%	4	2.35%	10°			1.59%
60°	2.18%	1.5	2.66%	2.5	3.10%	5	2.13%	20°			1.96%
70°	3.09%	1.6	2.87%	3	3.03%	6	1.88%	30°			2.37%
80°	3.16%	1.7	2.90%	3.5	3.13%	7	1.53%	40°			2.99%
90°	3.76%	1.8	2.43%	4	3.10%	8	1.73%	(0°, 0°, −5°, 5°)			1.88%
100°	3.57%	1.9	2.54%	4.5	3.19%	9	2.00%	(0°, 0°, −10°, 10°)			1.66%
110°	3.68%	2	3.14%	5	3.33%	10	2.18%	(0°, 0°, −20°, 20°)			1.85%
120°	3.66%	Hue		Translate				(0°, 0°, −30°, 30°)			2.32%
130°	3.56%							(0°, 0°, −40°, 40°)			2.75%
140°	3.32%	0.1	1.40%	(0.1, 0.1)			1.63%	(−5°, 5°, −5°, 5°)			2.02%
150°	3.17%	0.2	1.68%	(0.2, 0.2)			1.44%	(−10°, 10°, −10°, 10°)			2.14%
160°	3.74%	0.3	1.69%	(0.3, 0.3)			1.02%	(−20°, 20°, −20°, 20°)			2.30%
170°	3.93%	0.4	1.57%	(0.4, 0.4)			−0.66%	(−30°, 30°, −30°, 30°)			2.16%
180°	3.64%	0.5	1.62%	(0.5, 0.5)			−0.90%	(−40°, 40°, −40°, 40°)			2.11%

**Table 8:** Performance of DA methods on $\Gamma_{\mathrm{CSD}}^{{\mathrm{train}}^{50}}$. The impact of different DA methods and parameters on $\Gamma_{\mathrm{CSD}}^{{\mathrm{train}}^{50}}$ classification performance, which is very different from the impact on $\Gamma_{\mathrm{PAP}}^{\mathrm{train}}$ and $\Gamma_{\mathrm{CCZ}}^{\mathrm{train}}$, is displayed. The displayed percentage values describe the increase/decrease of mAA in percent. The value is color-coded from blue (increase in accuracy) over white (no effect) to orange (decrease in accuracy).

**Random Rotation**		**Brightness**		**Contrast**		**Saturation**		**Random** **VerticalFlip**		**Random** **HorizontalFlip**	
1°	−0.08%	0.1	−0.12%	0.1	−0.09%	0.1	0.07%	0.1	−1.95%	0.1	0.59%
2°	0.32%	0.2	0.06%	0.2	−0.14%	0.2	0.17%	0.2	−1.43%	0.2	0.46%
3°	0.63%	0.3	0.12%	0.3	−0.06%	0.3	0.42%	0.3	−1.52%	0.3	0.63%
4°	0.39%	0.4	−0.16%	0.4	−0.08%	0.4	0.63%	0.4	−1.39%	0.4	0.68%
5°	0.30%	0.5	−0.04%	0.5	−0.16%	0.5	0.62%	0.5	−1.69%	0.5	0.59%
6°	0.52%	0.6	0.27%	0.6	−0.06%	0.6	0.69%	0.6	−1.82%	0.6	0.57%
7°	0.42%	0.7	0.20%	0.7	0.18%	0.7	0.61%	0.7	−2.07%	0.7	0.56%
8°	0.34%	0.8	0.17%	0.8	−0.35%	0.8	0.77%	0.8	−2.49%	0.8	0.51%
9°	0.31%	0.9	−0.18%	0.9	−0.08%	0.9	0.75%	0.9	−2.80%	0.9	0.37%
10°	0.19%	1	−0.19%	1	−0.56%	1	0.90%	1	−8.53%	1	−0.30%
20°	−0.67%	1.1	−0.60%	1.3	−0.67%	1.5	1.18%	Shear			
30°	−0.98%	1.2	−0.67%	1.5	−0.25%	2	1.33%				
40°	−1.61%	1.3	−0.33%	1.8	0.02%	3	1.36%	5°			−0.14%
50°	−0.47%	1.4	0.19%	2	−0.25%	4	1.30%	10°			0.25%
60°	−0.88%	1.5	0.07%	2.5	−0.33%	5	1.22%	20°			−0.06%
70°	−1.87%	1.6	−0.40%	3	−0.08%	6	1.16%	30°			−0.42%
80°	−2.67%	1.7	0.03%	3.5	0.22%	7	1.26%	40°			−0.25%
90°	−2.35%	1.8	0.11%	4	0.04%	8	1.30%	(0°, 0°, −5°, 5°)			0.92%
100°	−2.79%	1.9	0.57%	4.5	0.59%	9	1.15%	(0°, 0°, −10°, 10°)			0.89%
110°	−2.57%	2	0.40%	5	1.13%	10	1.25%	(0°, 0°, −20°, 20°)			0.31%
120°	−2.94%	Hue		Translate				(0°, 0°, −30°, 30°)			0.19%
130°	−3.85%							(0°, 0°, −40°, 40°)			−0.10%
140°	−3.81%	0.1	0.53%	(0.1, 0.1)			−0.38%	(−5°, 5°, −5°, 5°)			0.37%
150°	−3.89%	0.2	0.27%	(0.2, 0.2)			−0.14%	(−10°, 10°, −10°, 10°)			0.55%
160°	−4.80%	0.3	0.16%	(0.3, 0.3)			−0.57%	(−20°, 20°, −20°, 20°)			0.14%
170°	−4.31%	0.4	−0.01%	(0.4, 0.4)			−2.02%	(−30°, 30°, −30°, 30°)			−0.37%
180°	−4.28%	0.5	0.10%	(0.5, 0.5)			−2.46%	(−40°, 40°, −40°, 40°)			−1.18%

**Table 9:** Performance of DA methods on $\Gamma_{\mathrm{CSD}}^{{\mathrm{train}}^{150}}$. The heatmap shows the DA methods and parameters impact on $\Gamma_{\mathrm{CSD}}^{{\mathrm{train}}^{150}}$ classification performance. The displayed percentage values describe the increase/decrease of mAA in percent. The value is color-coded from blue (increase in accuracy) over white (no effect) to orange (decrease in accuracy).

**Random Rotation**		**Brightness**		**Contrast**		**Saturation**		**Random** **VerticalFlip**		**Random** **HorizontalFlip**	
1°	0.35%	0.1	0.23%	0.1	0.25%	0.1	0.13%	0.1	−1.22%	0.1	0.26%
2°	0.55%	0.2	0.40%	0.2	0.46%	0.2	0.24%	0.2	−1.37%	0.2	0.53%
3°	0.74%	0.3	0.34%	0.3	0.55%	0.3	0.23%	0.3	−1.78%	0.3	0.50%
4°	0.09%	0.4	0.62%	0.4	0.53%	0.4	0.29%	0.4	−1.82%	0.4	0.44%
5°	0.55%	0.5	0.73%	0.5	0.62%	0.5	0.21%	0.5	−2.21%	0.5	0.37%
6°	0.75%	0.6	0.91%	0.6	0.37%	0.6	0.29%	0.6	−2.31%	0.6	0.65%
7°	0.83%	0.7	0.51%	0.7	0.90%	0.7	0.42%	0.7	−2.33%	0.7	0.47%
8°	0.55%	0.8	0.86%	0.8	0.59%	0.8	0.49%	0.8	−2.33%	0.8	0.32%
9°	0.84%	0.9	0.86%	0.9	0.18%	0.9	0.48%	0.9	−1.64%	0.9	0.57%
10°	−0.21%	1	0.81%	1	1.07%	1	0.49%	1	−8.28%	1	0.48%
20°	0.32%	1.1	0.91%	1.3	0.35%	1.5	0.70%	Shear			
30°	0.31%	1.2	1.18%	1.5	1.37%	2	0.81%				
40°	−0.16%	1.3	1.33%	1.8	0.87%	3	0.82%	5°			0.28%
50°	−0.18%	1.4	1.11%	2	1.00%	4	0.94%	10°			0.78%
60°	−0.14%	1.5	1.24%	2.5	0.83%	5	1.02%	20°			0.50%
70°	−0.08%	1.6	1.33%	3	0.93%	6	1.09%	30°			0.17%
80°	−0.64%	1.7	0.56%	3.5	0.75%	7	1.27%	40°			−0.11%
90°	−2.72%	1.8	0.96%	4	1.40%	8	1.31%	(0°, 0°, −5°, 5°)			0.56%
100°	−2.48%	1.9	0.96%	4.5	0.73%	9	1.31%	(0°, 0°, −10°, 10°)			−0.18%
110°	−3.08%	2	1.26%	5	1.08%	10	1.11%	(0°, 0°, −20°, 20°)			0.32%
120°	−2.34%	Hue		Translate				(0°, 0°, −30°, 30°)			0.31%
130°	−2.49%							(0°, 0°, −40°, 40°)			−0.27%
140°	−3.21%	0.1	0.60%	(0.1, 0.1)			0.13%	(−5°, 5°, −5°, 5°)			0.87%
150°	−3.02%	0.2	0.54%	(0.2, 0.2)			−1.52%	(−10°, 10°, −10°, 10°)			0.91%
160°	−2.56%	0.3	0.56%	(0.3, 0.3)			−0.62%	(−20°, 20°, −20°, 20°)			−0.72%
170°	−3.26%	0.4	0.61%	(0.4, 0.4)			−1.47%	(−30°, 30°, −30°, 30°)			−0.14%
180°	−4.57%	0.5	0.48%	(0.5, 0.5)			−0.79%	(−40°, 40°, −40°, 40°)			−0.06%

**Table 10:** DA combination policies.

DA Policy	Function of Each Policy
RBC_1	$X_{i}=f_{CT}\left(f_{RR}\left(x_{i},d=150^{{}^{\circ}}\right),b=1.9,c=5\right)$
RBC_2	$X_{i}=f_{CT}\left(f_{RR}\left(x_{i},d=150^{{}^{\circ}}\right),b=2,c=5\right)$
RBC_3	$X_{i}=f_{CT}\left(f_{RR}\left(x_{i},d=140^{{}^{\circ}}\right),b=2,c=5\right)$
RBC_4	$X_{i}=f_{CT}\left(f_{RR}\left(x_{i},d=140^{{}^{\circ}}\right),b=1.9,c=5\right)$
RBC_5	$X_{i}=f_{CT}\left(f_{RR}\left(x_{i},d=140^{{}^{\circ}}\right),b=2,c=4.5\right)$
RBCS	$X_{i}=f_{RA}(f_{CT}\left(f_{RR}\left(x_{i},d=150^{{}^{\circ}}\right),b=2,c=5\right),s=40^{{}^{\circ}}$)

**Table 11:** Performance of policies applied on $\Gamma_{\mathrm{PAP}}$ and $\Gamma_{\mathrm{CCZ}}$. The best performing policy is highlighted in yellow.

	AA_IP	AA_CP	RBC_1	RBC_2	RBC_3	RBC_4	RBC_5	RBCS
$\Gamma_{\mathrm{PAP}}^{{\mathrm{train}}^{50}}$	+9.59%	+9.43%	+10.63%	+10.45%	+10.49%	+10.38%	+10.86%	+10.57%
$\Gamma_{\mathrm{PAP}}^{{\mathrm{train}}^{100}}$	+5.48%	+5.07%	+6.32%	+6.35%	+6.26%	+6.07%	+5.86%	+6.14%
$\Gamma_{\mathrm{PAP}}^{{\mathrm{train}}^{200}}$	+3.19%	+2.89%	+3.73%	+3.66%	+3.72%	+3.80%	+3.61%	+3.66%
$\Gamma_{\mathrm{PAP}}^{\mathrm{train}}$	+2.48%	+2.66%	+2.76%	+2.84%	+2.80%	+2.91%	+2.83%	+2.88%
$\Gamma_{\mathrm{CCZ}}^{{\mathrm{train}}^{50}}$	+6.23%	+5.06%	+6.78%	+6.77%	+6.73%	+7.31%	+7.05%	+7.10%
$\Gamma_{\mathrm{CCZ}}^{{\mathrm{train}}^{100}}$	+4.26%	+3.81%	+4.52%	+4.08%	+4.68%	+4.21%	+4.74%	+4.58%
$\Gamma_{\mathrm{CCZ}}^{\mathrm{train}}$	+2.41%	+2.04%	+2.39%	+2.52%	+2.50%	+2.37%	+2.58%	+2.46%
$\Gamma_{\mathrm{CSD}}^{{\mathrm{train}}^{50}}$	+0.94%	+0.92%	−1.47%	−1.73%	−1.54%	−0.81%	−0.82%	−2.67%
$\Gamma_{\mathrm{CSD}}^{{\mathrm{train}}^{100}}$	+2.57%	+1.06%	−2.61%	−1.95%	−1.99%	−2.12%	−1.80%	−1.53%
$\Gamma_{\mathrm{CSD}}^{\mathrm{train}}$	+1.17%	+1.44%	−1.02%	−1.76%	−1.31%	−2.12%	−1.08%	−1.65%

**Figure A1:**
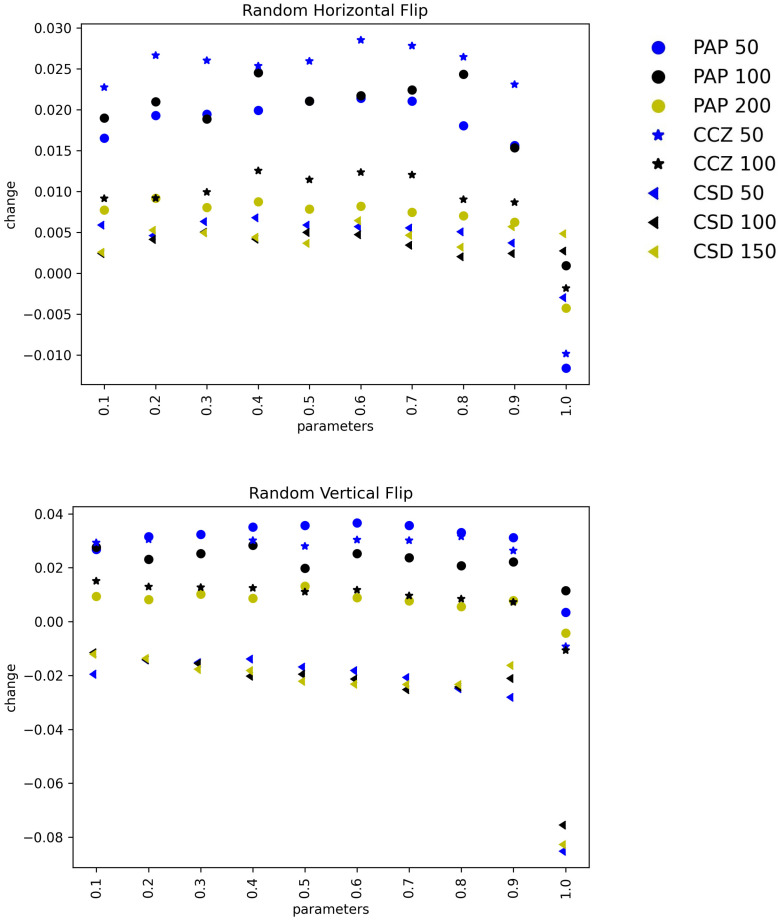
Impacts comparison of Random Horizontal Flip and Random Vertical Flip. The figure reveals the impacts of the different parameters of Random Horizontal Flip and Random Vertical Flip on $\Gamma_{\mathrm{PAP}}$, $\Gamma_{\mathrm{CCZ}}$ and $\Gamma_{\mathrm{CSD}}$ classification mAA improvement.

**Figure A2:**
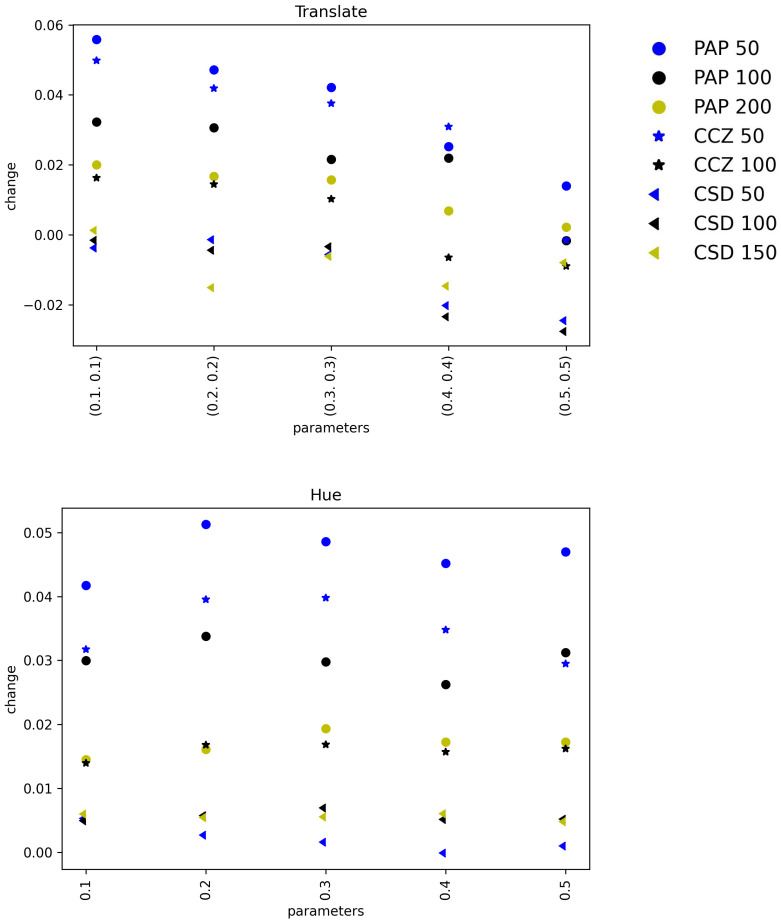
Impacts comparison of Translate and Hue. The figure shows the performance of the different parameters of Translate and Hue on the three datasets.

## Data Availability

The data are available on request from the corresponding author.

## Abbreviations

The following abbreviations are used in this manuscript:

AUV	Autonomous Underwater Vehicles
ROV	Remotely Operated Vehicles
SVM	Support Vector Machines
CNN	Convolutional Neural Networks
DA	Data Augmentation
GAN	Generative Adversarial Networks
PAP	Porcupine Abyssal Plane
CCZ	Clarion Clipperton Zone
CSD	Cityscapes Dataset

## Appendix A. Performance of DA Methods on $\Gamma_{\mathrm{PAP}}^{{\mathrm{train}}^{100}}$ and $\Gamma_{\mathrm{CSD}}^{{\mathrm{train}}^{100}}$

**Table A1:** Performance of DA methods on $\Gamma_{\mathrm{PAP}}^{{\mathrm{train}}^{100}}$. This table indicates the augment effect of different DA methods and parameters applied on $\Gamma_{\mathrm{PAP}}^{{\mathrm{train}}^{100}}$ when setting seed to 350. The displayed percentage values describe the increase/decrease of mAA in percent. The value is color-coded from blue (increase in accuracy) over white (no effect) to orange (decrease in accuracy). The four DA methods with the best effect are still RandomRotation, Brightness, Contrast, and Shear. These four methods can obtain their best enhancement effect on $\Gamma_{\mathrm{PAP}}^{{\mathrm{train}}^{100}}$ with parameters 160°, 2, 5, and (−20°, 20°, −20°, 20°), respectively. The trend of the effect of different parameters is similar to that of $\Gamma_{\mathrm{PAP}}^{{\mathrm{train}}^{50}}$, but with fewer increments for the average accuracy.

**Random Rotation**		**Brightness**		**Contrast**		**Saturation**		**Random** **VerticalFlip**		**Random** **HorizontalFlip**	
1°	2.64%	0.1	2.86%	0.1	2.27%	0.1	1.72%	0.1	2.76%	0.1	1.90%
2°	3.37%	0.2	2.82%	0.2	2.10%	0.2	2.73%	0.2	2.31%	0.2	2.10%
3°	3.40%	0.3	3.57%	0.3	3.17%	0.3	2.70%	0.3	2.52%	0.3	1.89%
4°	3.37%	0.4	3.34%	0.4	2.70%	0.4	2.32%	0.4	2.83%	0.4	2.45%
5°	3.34%	0.5	4.02%	0.5	3.23%	0.5	2.41%	0.5	1.98%	0.5	2.11%
6°	3.79%	0.6	4.22%	0.6	4.02%	0.6	2.92%	0.6	2.52%	0.6	2.17%
7°	3.13%	0.7	4.43%	0.7	3.46%	0.7	2.00%	0.7	2.37%	0.7	2.24%
8°	3.73%	0.8	4.03%	0.8	3.51%	0.8	2.75%	0.8	2.07%	0.8	2.44%
9°	2.96%	0.9	4.11%	0.9	4.84%	0.9	3.15%	0.9	2.21%	0.9	1.54%
10°	2.98%	1	4.65%	1	4.45%	1	3.16%	1	1.14%	1	0.09%
20°	3.66%	1.1	4.69%	1.3	4.33%	1.5	3.68%	Shear			
30°	4.06%	1.2	4.17%	1.5	4.54%	2	2.97%				
40°	4.71%	1.3	4.41%	1.8	4.48%	3	3.48%	5°			2.01%
50°	4.94%	1.4	4.91%	2	4.65%	4	3.62%	10°			3.30%
60°	5.21%	1.5	4.91%	2.5	4.37%	5	3.58%	20°			3.45%
70°	4.83%	1.6	4.74%	3	4.50%	6	4.03%	30°			4.14%
80°	4.49%	1.7	5.08%	3.5	4.11%	7	4.05%	40°			3.26%
90°	5.07%	1.8	4.79%	4	4.50%	8	4.17%	(0°, 0°, −5°, 5°)			2.91%
100°	5.00%	1.9	4.38%	4.5	4.88%	9	3.39%	(0°, 0°, −10°, 10°)			3.31%
110°	5.37%	2	5.08%	5	5.40%	10	3.94%	(0°, 0°, −20°, 20°)			3.37%
120°	4.72%	Hue		Translate				(0°, 0°, −30°, 30°)			3.20%
130°	4.92%							(0°, 0°, −40°, 40°)			4.27%
140°	4.80%	0.1	3.00%	(0.1, 0.1)			3.23%	(−5°, 5°, −5°, 5°)			2.77%
150°	4.76%	0.2	3.38%	(0.2, 0.2)			3.06%	(−10°, 10°, −10°, 10°)			3.81%
160°	5.61%	0.3	2.98%	(0.3, 0.3)			2.16%	(−20°, 20°, −20°, 20°)			4.54%
170°	5.43%	0.4	2.63%	(0.4, 0.4)			2.19%	(−30°, 30°, −30°, 30°)			4.03%
180°	5.17%	0.5	3.12%	(0.5, 0.5)			−0.17%	(−40°, 40°, −40°, 40°)			4.45%

**Table A2:** Performance of DA methods on $\Gamma_{\mathrm{CSD}}^{{\mathrm{train}}^{100}}$. This heatmap shows the results when we increase the training set samples to 100 per class. The displayed percentage values describe the increase/decrease of mAA in percent. The value is color-coded from blue (increase in accuracy) over white (no effect) to orange (decrease in accuracy). We can see that RandomRotation and RandomVerticalFlip have a negative impact. Saturation with a parameter of 9 and Brightness with a parameter of 1.7 are the two most effective DA methods and parameters.

**Random Rotation**		**Brightness**		**Contrast**		**Saturation**		**Random** **VerticalFlip**		**Random** **HorizontalFlip**	
1°	0.14%	0.1	0.13%	0.1	0.10%	0.1	0.09%	0.1	−1.16%	0.1	0.24%
2°	0.15%	0.2	0.23%	0.2	0.05%	0.2	0.08%	0.2	−1.42%	0.2	0.41%
3°	0.23%	0.3	0.23%	0.3	0.08%	0.3	0.18%	0.3	−1.56%	0.3	0.50%
4°	0.05%	0.4	0.31%	0.4	0.16%	0.4	0.15%	0.4	−2.02%	0.4	0.42%
5°	0.38%	0.5	0.26%	0.5	0.22%	0.5	0.26%	0.5	−1.95%	0.5	0.50%
6°	0.51%	0.6	0.27%	0.6	0.25%	0.6	0.07%	0.6	−2.13%	0.6	0.47%
7°	0.50%	0.7	−0.08%	0.7	−0.23%	0.7	0.08%	0.7	−2.53%	0.7	0.34%
8°	0.65%	0.8	0.03%	0.8	−1.36%	0.8	0.30%	0.8	−2.43%	0.8	0.20%
9°	0.55%	0.9	0.60%	0.9	0.13%	0.9	0.21%	0.9	−2.10%	0.9	0.24%
10°	−0.01%	1	0.66%	1	0.19%	1	0.24%	1	−7.55%	1	0.27%
20°	−0.01%	1.1	0.88%	1.3	0.41%	1.5	0.37%	Shear			
30°	−0.17%	1.2	0.71%	1.5	0.50%	2	0.53%				
40°	−0.20%	1.3	0.45%	1.8	0.45%	3	0.63%	5°			0.36%
50°	0.10%	1.4	0.29%	2	0.62%	4	1.02%	10°			0.44%
60°	−0.45%	1.5	0.75%	2.5	0.70%	5	1.27%	20°			0.11%
70°	−0.56%	1.6	0.70%	3	0.52%	6	1.14%	30°			−0.03%
80°	−3.18%	1.7	0.95%	3.5	−0.31%	7	1.24%	40°			0.01%
90°	−2.44%	1.8	0.57%	4	0.31%	8	1.30%	(0°, 0°, −5°, 5°)			0.29%
100°	−2.68%	1.9	0.58%	4.5	0.83%	9	1.41%	(0°, 0°, −10°, 10°)			0.33%
110°	−2.11%	2	0.32%	5	−0.36%	10	1.35%	(0°, 0°, −20°, 20°)			0.28%
120°	−2.34%	Hue		Translate				(0°, 0°, −30°, 30°)			0.48%
130°	−1.83%							(0°, 0°, −40°, 40°)			0.56%
140°	−3.11%	0.1	0.50%	(0.1, 0.1)			−0.16%	(−5°, 5°, −5°, 5°)			0.53%
150°	−3.36%	0.2	0.57%	(0.2, 0.2)			−0.44%	(−10°, 10°, −10°, 10°)			0.37%
160°	−3.38%	0.3	0.69%	(0.3, 0.3)			−0.34%	(−20°, 20°, −20°, 20°)			0.50%
170°	−3.22%	0.4	0.52%	(0.4, 0.4)			−2.34%	(−30°, 30°, −30°, 30°)			0.35%
180°	−3.12%	0.5	0.52%	(0.5, 0.5)			−2.76%	(−40°, 40°, −40°, 40°)			−0.21%

## Appendix B. Performance of DA Methods on $\Gamma_{\mathrm{PAP}}^{{\mathrm{train}}^{50}}$ and $\Gamma_{\mathrm{CCZ}}^{{\mathrm{train}}^{50}}$ with Setting Seed to 3500

**Table A3:** Performance of DA methods on $\Gamma_{\mathrm{PAP}}^{{\mathrm{train}}^{50}}$ with setting seed to 3500. This heatmap shows the effect of different DA methods and parameters on the average accuracy improvement of $\Gamma_{\mathrm{PAP}}^{{\mathrm{train}}^{50}}$ when setting seed to 3500. The displayed percentage values describe the increase/decrease of mAA in percent. The value is color-coded from blue (increase in accuracy) over white (no effect) to orange (decrease in accuracy). DA methods show a similar trend to that at seed is 350.

**Random Rotation**		**Brightness**		**Contrast**		**Saturation**		**Random** **VerticalFlip**		**Random** **HorizontalFlip**	
1°	3.26%	0.1	1.76%	0.1	1.06%	0.1	0.75%	0.1	2.26%	0.1	1.60%
2°	4.34%	0.2	2.57%	0.2	1.52%	0.2	0.88%	0.2	2.64%	0.2	1.75%
3°	4.96%	0.3	3.19%	0.3	2.12%	0.3	1.32%	0.3	2.87%	0.3	2.00%
4°	5.21%	0.4	3.16%	0.4	1.24%	0.4	1.63%	0.4	2.91%	0.4	2.08%
5°	5.45%	0.5	3.59%	0.5	2.98%	0.5	1.63%	0.5	2.99%	0.5	1.97%
6°	5.58%	0.6	3.94%	0.6	3.29%	0.6	1.94%	0.6	2.79%	0.6	1.93%
7°	5.65%	0.7	4.16%	0.7	3.61%	0.7	2.16%	0.7	2.72%	0.7	1.88%
8°	5.68%	0.8	4.70%	0.8	4.01%	0.8	2.11%	0.8	2.74%	0.8	1.67%
9°	5.75%	0.9	5.30%	0.9	4.43%	0.9	2.51%	0.9	2.55%	0.9	1.21%
10°	5.58%	1	5.76%	1	4.84%	1	2.48%	1	−0.51%	1	−0.36%
20°	5.61%	1.1	5.34%	1.3	4.83%	1.5	2.69%	Shear			
30°	5.81%	1.2	5.34%	1.5	4.83%	2	3.00%				
40°	6.10%	1.3	6.17%	1.8	4.77%	3	3.17%	5°			3.36%
50°	6.25%	1.4	5.97%	2	5.12%	4	3.50%	10°			3.68%
60°	6.25%	1.5	6.10%	2.5	5.78%	5	4.02%	20°			4.30%
70°	6.71%	1.6	5.92%	3	5.49%	6	4.20%	30°			5.79%
80°	7.21%	1.7	6.06%	3.5	5.80%	7	4.25%	40°			5.65%
90°	7.31%	1.8	5.66%	4	5.75%	8	4.22%	(0°, 0°, −5°, 5°)			4.17%
100°	7.59%	1.9	6.13%	4.5	5.12%	9	4.48%	(0°, 0°, −10°, 10°)			4.60%
110°	7.45%	2	6.34%	5	6.70%	10	4.53%	(0°, 0°, −20°, 20°)			4.98%
120°	7.21%	Hue		Translate				(0°, 0°, −30°, 30°)			5.59%
130°	7.24%							(0°, 0°, −40°, 40°)			5.98%
140°	7.10%	0.1	3.01%	(0.1, 0.1)			5.50%	(−5°, 5°, −5°, 5°)			5.32%
150°	7.53%	0.2	3.91%	(0.2, 0.2)			4.20%	(−10°, 10°, −10°, 10°)			5.07%
160°	7.28%	0.3	3.83%	(0.3, 0.3)			3.31%	(−20°, 20°, −20°, 20°)			5.52%
170°	7.42%	0.4	3.75%	(0.4, 0.4)			1.54%	(−30°, 30°, −30°, 30°)			6.32%
180°	6.94%	0.5	3.47%	(0.5, 0.5)			−3.24%	(−40°, 40°, −40°, 40°)			5.41%

**Table A4:** Performance of DA methods on $\Gamma_{\mathrm{CCZ}}^{{\mathrm{train}}^{50}}$ with setting seed to 3500. The displayed percentage values describe the increase/decrease of mAA in percent. The value is color-coded from blue (increase in accuracy) over white (no effect) to orange (decrease in accuracy). This heatmap shows the effect of different DA methods and different parameters on the average accuracy improvement of $\Gamma_{\mathrm{CCZ}}^{{\mathrm{train}}^{50}}$ when setting seed to 3500. As with the experimental results on $\Gamma_{\mathrm{PAP}}^{{\mathrm{train}}^{50}}$, the performance of DA is similar under different seeds.

**Random Rotation**		**Brightness**		**Contrast**		**Saturation**		**Random** **VerticalFlip**		**Random** **HorizontalFlip**	
1°	4.14%	0.1	2.76%	0.1	2.45%	0.1	1.51%	0.1	3.61%	0.1	2.72%
2°	4.11%	0.2	3.18%	0.2	3.23%	0.2	1.62%	0.2	3.20%	0.2	3.02%
3°	4.60%	0.3	4.03%	0.3	3.23%	0.3	1.21%	0.3	3.23%	0.3	3.10%
4°	4.56%	0.4	4.08%	0.4	3.93%	0.4	2.08%	0.4	3.10%	0.4	3.28%
5°	4.53%	0.5	4.49%	0.5	4.25%	0.5	2.10%	0.5	3.14%	0.5	3.04%
6°	4.51%	0.6	4.75%	0.6	4.42%	0.6	2.12%	0.6	3.11%	0.6	3.12%
7°	4.43%	0.7	4.35%	0.7	4.59%	0.7	2.34%	0.7	2.90%	0.7	3.23%
8°	4.69%	0.8	4.47%	0.8	4.66%	0.8	2.00%	0.8	2.57%	0.8	3.10%
9°	4.97%	0.9	4.86%	0.9	4.79%	0.9	2.11%	0.9	2.32%	0.9	2.81%
10°	4.61%	1	4.58%	1	3.44%	1	2.22%	1	−1.51%	1	0.16%
20°	5.28%	1.1	4.45%	1.3	5.09%	1.5	2.73%	Shear			
30°	5.16%	1.2	4.83%	1.5	4.50%	2	2.71%				
40°	5.22%	1.3	5.08%	1.8	4.85%	3	2.83%	5°			4.18%
50°	5.44%	1.4	4.86%	2	5.29%	4	2.93%	10°			4.38%
60°	5.58%	1.5	5.33%	2.5	5.64%	5	2.93%	20°			4.56%
70°	5.76%	1.6	5.20%	3	5.75%	6	3.09%	30°			4.95%
80°	5.50%	1.7	4.93%	3.5	5.21%	7	2.78%	40°			5.21%
90°	6.19%	1.8	4.86%	4	5.82%	8	2.52%	(0°, 0°, −5°, 5°)			4.13%
100°	6.31%	1.9	4.89%	4.5	5.38%	9	2.60%	(0°, 0°, −10°, 10°)			4.49%
110°	6.27%	2	4.81%	5	5.48%	10	2.41%	(0°, 0°, −20°, 20°)			4.22%
120°	5.73%	Hue		Translate				(0°, 0°, −30°, 30°)			5.00%
130°	5.82%							(0°, 0°, −40°, 40°)			4.77%
140°	6.08%	0.1	2.59%	(0.1, 0.1)			4.30%	(−5°, 5°, −5°, 5°)			4.98%
150°	5.97%	0.2	2.77%	(0.2, 0.2)			4.07%	(−10°, 10°, −10°, 10°)			4.97%
160°	6.07%	0.3	2.79%	(0.3, 0.3)			3.54%	(−20°, 20°, −20°, 20°)			5.29%
170°	5.98%	0.4	3.35%	(0.4, 0.4)			1.94%	(−30°, 30°, −30°, 30°)			4.84%
180°	6.22%	0.5	3.12%	(0.5, 0.5)			−1.62%	(−40°, 40°, −40°, 40°)			4.72%
